# Task-Offloading Optimization Using a Genetic Algorithm in Hybrid Fog Computing for the Internet of Drones

**DOI:** 10.3390/s25051383

**Published:** 2025-02-24

**Authors:** Mohamed Amine Attalah, Sofiane Zaidi, Naçima Mellal, Carlos T. Calafate

**Affiliations:** 1 Department of Electronics, University Center of Tipaza, Tipaza 42000, Algeria; attalah.mohamed@cu-tipaza.dz; 2 Department of Mathematics and Computer Science, Research Laboratory on Computer Science’s Complex Systems (RELA(CS)2), University of Oum El Bouaghi, Oum El Bouaghi 04000, Algeria; zaidi.sofiane@univ-oeb.dz; 3 Department of Networks & Telecommunications, Research Laboratory of Artificial Intelligence and Autonomous Objects (LAIAO), University of Oum El Bouaghi, Oum El Bouaghi 04000, Algeria; nassima.mellal@univ-oeb.dz; 4 Department of Computer Engineering (DISCA), Universitat Politècnica de València, 46022 Valencia, Spain

**Keywords:** Internet of Drones, fog computing networks, genetic algorithm optimization, task offloading in IoD, unmanned aerial vehicles

## Abstract

Research and development on task offloading over the Internet of Drones (IoD) has expanded rapidly in the last few years. Task offloading in a fog IoD environment is very challenging due to the high dynamics of the IoD topology, which cause intermittent connections, as well as the stringent requirements of task offloading, such as reduced delay. To overcome these challenges, in this paper, we propose a task-offloading optimization strategy using a heuristic genetic algorithm (GA) with hybrid fog computing technology for the Internet of Drones, named GA Hybrid-Fog. The proposed solution employs a GA for task offloading from edge Unmanned Aerial Vehicles (UAVs) to both fog base stations (FBSs) and fog UAVs (FUAVs) in order to optimize offloading delays (transmission and fog computing delays) and guarantee higher storage and processing capacity. Experimental results show that GA Hybrid-Fog achieves greater improvements in task-offloading delays compared to other IoD technologies (GA BS-Fog, GA UAV-Fog, and GA UAV-Edge).

## 1. Introduction

The Internet of Drones (IoD) is a new paradigm in which a set of flying nodes, known as drones or Unmanned Aerial Vehicles (UAVs), communicate with one another or with ground infrastructure via the Internet to perform various tasks, such as environmental monitoring, video streaming, surveillance, smart agriculture, urban planning, object tracking, and disaster management [1].

IoD standards facilitate the regulation of various solutions for flying objects developed by different manufacturers, enabling them to communicate with one another across various hardware architectures, platforms, and communication protocols. Several IoD standardization initiatives have been promoted by various industry and government standardization organizations, as well as specific interest groups, including the Third Generation Partnership Project (3GPP) [2], the International Telecommunication Union Telecommunication Standardization Sector (ITU-T) [3], and the European Telecommunications Standards Institute (ETSI) [4].

The IoD offers many advantages by integrating drones with the Internet of Things (IoT), such as extending connectivity and coverage; increasing reliability, storage, and processing capacity; improving resistance to weather conditions; and enhancing energy supply for IoT nodes [5].

IoD technologies can be classified into four distinct categories: IoD cloud computing, IoD edge computing, IoD fog computing, and IoD cellular networks [6]. IoD cloud computing provides significant computational and storage capacity for drone-collected data by transferring the data to cloud servers. In contrast, IoD edge and fog computing technologies aim to reduce data transmission latency by enabling local processing and storage of UAV-collected data at edge and fog nodes within the network [7,8].

The IoD cellular network is regarded as a highly promising technology for real-time applications due to its exceptional reliability, high data rates, and low latency. Moreover, IoD cellular networks can significantly improve IoD performance in various aspects, including connectivity, accessibility, monitoring, management, navigation, and cost efficiency [9].

The task-offloading mechanism aims to distribute tasks among resource nodes while jointly allocating communication, computation, and storage resources. According to [10], task offloading typically relies on two parameters. The first parameter efficiently solves a multi-objective optimization problem to determine an optimal operating point, while the second parameter focuses on the dynamic nature of task offloading. Theoretical analysis methods and stability controllers have been developed to ensure robustness against rapid changes in parameters, resource availability, and network conditions [11].

In resource management systems, offloading and scheduling are complementary but distinct processes that play crucial roles in optimizing system performance. Task offloading refers to the transfer of computational tasks from resource-constrained devices (such as drones in the IoD) to more capable computing nodes, such as edge servers, fog base stations, or the cloud. This process aims to reduce the computational load on the local device and minimize processing delays, energy consumption, or both. In contrast, task scheduling involves determining the order, priority, and resource allocation for tasks either at the source device or the receiving node (e.g., fog or edge server) to optimize the use of available resources, such as CPU, memory, and network bandwidth. Some works consider both task offloading and scheduling in the IoD; for instance, the authors of [12] proposed a model in which tasks are not only offloaded to edge servers but also scheduled based on their dependencies and deadlines. This combined approach ensures the timely execution of tasks while balancing the computational load across the network.

### 1.1. Motivation

Existing task-offloading schemes and frameworks in IoD fog computing rely on stationary fog nodes (base stations) or mobile fog nodes (drones). The first category allows for higher storage and computing capacity, which helps reduce processing delays, while the second category achieves reduced transmission delays since the fog UAVs are near the edge nodes. Nevertheless, fog BS-based platforms are not suitable for real-time applications due to higher transmission delays, and the UAV-Fog method is limited in applications that require higher processing capacity due to the limited resources of fog UAVs. Based on the aforementioned gaps in the literature, we take advantage of both fog BS and fog UAV elements by proposing a hybrid task-offloading system for IoD fog computing. Moreover, our platform employs a heuristic genetic algorithm to find the optimal offloading strategy that minimizes both transmission and computing delays.

### 1.2. Contribution

In IoD networks, minimizing task-offloading delays from edge UAVs to fog base stations (FBSs) and fog UAVs presents a significant challenge due to the limited available resources of UAVs. To address this challenge, in this paper, we propose a task-offloading solution called GA Hybrid-Fog that combines fog BSs, fog UAVs, and a GA to optimize both transmission and fog computing delays. Specifically, GA Hybrid-Fog allows onboard computing resources of edge UAVs to be integrated with the fog computing environment, which consists of stationary FBS and mobile fog UAV (FUAV) computational entities. Moreover, this solution reaps the benefits of the fog computing capabilities of the base stations and UAVs, including processing, storage, and sensing, to enhance traditional fog computing capabilities with UAVs.

### 1.3. Organization

The rest of this paper is organized as follows. Section 2 presents a literature review. Section 3 introduces IoD technologies and highlights our proposed GA Hybrid-Fog model, including the architecture, mathematical formulation, and algorithms. Section 4 describes the experimental study and analyzes the numerical results. Finally, Section 5 concludes this paper with a summary and suggests potential future research directions.

## 2. Related Works

In the IoD fog computing literature, many solutions have been proposed. However, they are limited in demonstrating the effectiveness of fog computing for task offloading in the IoD compared with IoD cloud computing and IoD edge computing approaches that use a metaheuristic method, such as particle swarm optimization, genetic algorithms (GAs), or others.

Among the existing works on this topic, S. Zaidi et al. proposed a PSO (particle swarm optimization)-based task-offloading solution for fog computing in IoT applications, named PSO BS-Fog [13]. This solution allows a set of UAVs to offload their tasks to fog base stations (FBSs) for processing and storage. The simulation results demonstrated that the proposed solution achieved a lower offloading delay compared to PSO UAV-Fog and PSO UAV-Edge. However, the offloading delay of PSO BS-Fog can be further improved by integrating UAV-Fog, which can process and store offloaded tasks of appropriate size. Furthermore, utilizing another metaheuristic algorithm, such as a genetic algorithm (GA), could significantly optimize the task-offloading delay compared to PSO.

In [14], M. Aldossary proposed a collaborative strategy for task offloading in IoD fog computing. Moreover, the author also proposed using the mixed-integer linear programming (MILP) model to formulate the task-offloading problem to optimize both UAV energy consumption and offloading latency. The simulation results confirmed that the proposed model provides lower energy consumption and lower offloading delay compared to IoD cloud computing.

In [15], G. Sun et al. presented an architecture for task offloading in the IoD based on three layers: a fog layer, a UAV client layer, and a UAV edge layer. Moreover, the authors proposed optimizing both task offloading and resource allocation in this architecture using a game-theoretic algorithm and a convex optimization-based algorithm, respectively. The experimental results showed that the proposed approach achieved better performance under heavy system workloads.

F. S. Abkenar proposed a framework for disaster management based on IoD fog computing [16]. Moreover, the author developed three algorithms to optimize UAV energy consumption. The first algorithm optimizes the UAV’s position, the second algorithm optimizes task offloading to fog UAVs, and the third algorithm is based on wireless power transfer technology and supplies the UAV’s battery when its energy drops below a predefined threshold. The numerical results confirmed that the proposed framework achieves better performance in terms of energy consumption, delay, and throughput.

In [17], W. Min et al. proposed a task-offloading model based on dynamic programming for IoD fog computing. For task-offloading optimization, the proposed model considers drone mobility, fog node processing capacity, IoT communication constraints, and latency. Simulation experiments showed that the proposed model significantly improves task offloading in terms of latency, availability, and cost of resources.

Y. Jingjing and A. Nirwan [18] proposed optimizing task offloading and power consumption in IoD fog computing using machine learning (ML). For each drone, the proposed approach determines the number of offloaded images and the wireless transmission power required.

In [19], Z. Qingyang et al. investigated the performance of an IoD fog computing system consisting of many drones that offloaded their tasks to a fog node. Moreover, based on Stochastic Reward Nets (SRNs), the authors analyzed the effect of the number of offloaded tasks on the performance bottleneck of the drone computation system. Simulation experiments demonstrated that task-offloading performance deteriorated when the number of competing drones increased.

In [20], S. Tong et al. proposed a UAV-based multi-hop collaborative model for task offloading in fog computing. In the model, a set of fog UAVs is deployed to provide computing services to ground users. Moreover, the authors proposed formulating the task-offloading problem as a mixed-integer nonlinear programming (MINLP) optimization problem that takes into account parameters such as UAV association, task offloading, transmission power, resource allocation, and UAV location. The simulation results demonstrated the effectiveness of the proposed model.

A. Naouri et al. [21] evaluated the Whale Optimization Algorithm (WOA) for UAV fog node deployment in critical rescue operations and compared it against other optimization techniques, such as PSO, Harris Hawks Optimization (HHO), the Sine Cosine Algorithm (SCA), the Slime Mold Algorithm (SMA), and accelerated particle swarm optimization (APSO). The experimental results demonstrated that the WOA algorithm outperformed other algorithms in terms of connectivity, coverage, stability, and efficiency.

In [22], B. Liu et al. introduced the Hybrid Multi-Strategy Artificial Rabbits Optimization (HARO) algorithm to enhance UAV path planning in complex environments such as urban and mountainous areas. Conventional algorithms often face challenges like high computational costs and the tendency to become trapped in local optima. HARO overcomes these limitations by employing a dual exploration switching strategy and a population migration memory mechanism to balance exploration and exploitation. Additionally, it incorporates spherical and cylindrical obstacle models for realistic simulations and utilizes a key-point retention strategy to eliminate redundant waypoints, thereby reducing flight costs. The experimental results demonstrated that HARO improves search performance, enabling more efficient and stable UAV navigation.

Table 1 presents a comparative analysis of various studies on IoD fog computing based on various criteria, including the nature of fog nodes, objectives, addressed problems, optimization techniques, and application domains, which are described as follows:

- Nature of fog nodes: This criterion represents the task-offloading strategy of the proposed IoD fog computing platform, which may involve stationary fog nodes, UAV-based fog nodes, or a combination of both.
- Objectives: Each study focuses on different objectives, such as minimizing real-time latency, optimizing energy consumption, improving system utility, and enhancing service availability.
- Addressed problems: These include task offloading, resource allocation, UAV deployment, connectivity, and coverage, among others.
- Optimization techniques: Various optimization methods can be employed, including particle swarm optimization (PSO), mixed-integer linear programming (MILP), game-theoretic approaches, convex optimization, genetic algorithms (GAs), dynamic programming, convolutional neural networks (CNNs), mixed-integer nonlinear programming (MINLP), and stochastic routing networks (SRNs).
- Application domains: Some studies focus on general fog computing functions such as processing, storage, and networking, while others target specific applications like post-disaster rescue, disaster management, object recognition, and critical rescue operations.

The table shows that existing studies rely on either stationary fog nodes or UAV-based fog nodes. However, our proposed approach uniquely integrates stationary and mobile fog nodes, leveraging their combined advantages. Moreover, the table highlights that real-time latency optimization and task offloading are the most critical challenges and objectives in IoD fog computing. Therefore, our work focuses on addressing these challenges. Furthermore, in our work, we employ a GA instead of the metaheuristic approaches used in other IoD fog computing studies, as prior research has demonstrated the superior effectiveness of GAs. Adopting a hybrid fog computing architecture enhances processing, storage, and energy capacity, making it well suited for GA-based optimization.

## 3. GA Hybrid-Fog Model

This paper introduces the GA Hybrid-Fog model, a novel fog computing framework designed to enhance the capabilities of IoD systems. Unlike traditional IoD infrastructures that primarily rely on cloud computing, fog base stations, or edge computing nodes, GA Hybrid-Fog integrates multiple computing resources, including FBSs, fog UAVs, edge UAVs, and cloud computing. By leveraging the onboard computing capabilities of UAVs and the resources of FBSs, the GA Hybrid-Fog model significantly expands the available computational capacity.

The GA Hybrid-Fog model allows drones to seamlessly access the computing resources of both mobile and stationary fog nodes, thereby improving computational efficiency. Furthermore, it reduces latency by enabling direct interactions between UAVs and fog UAVs, FBSs, and cloud platforms. Structured with four distinct layers—an edge layer, a mobile fog layer, a static fog layer, and a cloud layer—the proposed model enhances reliability and minimizes latency, particularly in real-time applications. Additionally, it supports a wide range of computing functions, including data processing, storage, and networking.

### 3.1. IoD Technologies

The rapid evolution of UAV technology allows for its integration with diverse networks and systems to complete complex missions. To improve the availability of IoD services, IoD systems must effectively leverage current technologies [23]. Below, we explore the most significant and recent technologies employed in IoD networks:

- IoD Cloud Computing Technology

Given the limitations in wireless communication and the computing capabilities of drones, cloud computing is well suited for IoD systems. Specifically, cloud computing offers data processing and storage services for UAVs. As a result, UAV devices do not need to be highly powerful in terms of CPU speed and memory capacity, since complex computations can be handled by the cloud [24].

Cloud computing offers numerous advantages, including extended battery life, increased storage, scalability, and reliability. However, it also faces challenges, such as security and privacy concerns, bandwidth and data transfer issues, data management and synchronization difficulties, and energy efficiency problems [25].

- IoD Fog Computing Technology

Due to the limited computing resources on drones, managing computationally intensive tasks locally is challenging, which often leads to the use of cloud-based offloading. However, cloud computing solutions may be inadequate for applications requiring low latency and high reliability. This is mostly due to the slow response times resulting from long-distance data transmission. To address these challenges, fog computing has been proposed [26]. It offers an intermediate layer positioned at the edge of the network between UAVs and the cloud, which comprises numerous fog nodes. This fog layer connects to the cloud layer via the Internet or communicates with UAVs through a wireless connection.

- IoD Edge Computing Technology

IoD edge computing enhances fog and cloud computing capabilities for real-time IoT applications. By handling some UAV data locally at edge IoD devices, the edge layer reduces the computing load without relying on fog or cloud interventions. This shift to edge-based data processing and storage significantly improves latency. Recent research on the IoD has increasingly focused on edge computing to support real-time applications, such as smart transportation, video streaming, surveillance, augmented reality, emergency response, etc. [27]. In the proposed system, tasks are offloaded to edge UAVs using a GA. The UAVs are selected based on their proximity and lower processing frequency to ensure both low latency and high reliability.

### 3.2. GA Hybrid-Fog Architecture

**Figure 1:**
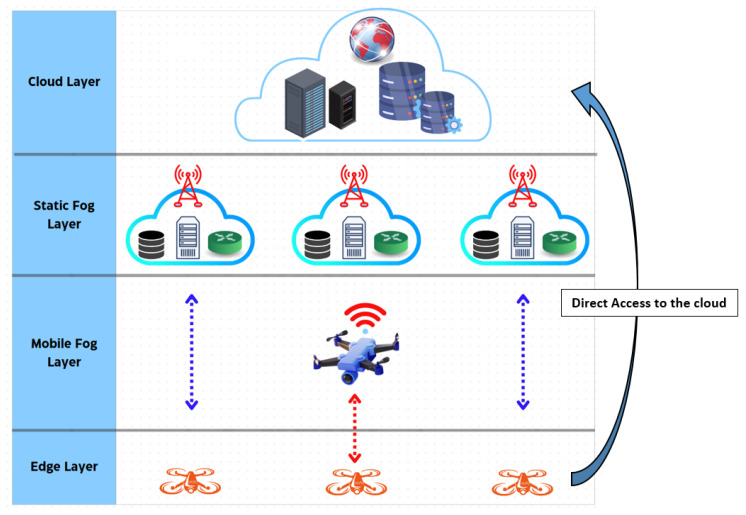
Architecture of the proposed GA Hybrid-Fog algorithm.

In our proposed model, all available computing resources (static fog, mobile fog, and cloud) are accessible to edge UAVs through either a direct or hierarchical method. The GA Hybrid-Fog algorithm is a cross-layer model that integrates infrastructure components, as illustrated in Figure 1. The components are described as follows:

- Edge Layer:

The edge layer is the lowest level in the architecture of the GA Hybrid-Fog algorithm and is the one closest to UAVs. Its primary role is to collect sensor data in the aerial environment, such as video, temperature, pressure, gas, etc. The collected data are either processed locally or transmitted to the fog UAVs, FBSs, or the cloud, depending on the size of the task and the required processing capabilities.

- Mobile Fog Layer:

The next layer in the GA Hybrid-Fog architecture is the mobile fog layer. It is characterized by higher computing and storage capabilities than edge UAVs. This architecture facilitates the processing of tasks offloaded from UAVs, effectively addressing the resource limitations of edge UAVs. The mobile fog layer communicates with UAVs and FBSs wirelessly. Additionally, in this layer, fog UAVs transmit and receive collected data via mobile technologies, including WiFi, WiMAX, and others.

- Static Fog Layer:

This layer serves as an intermediary between the mobile fog layer and the cloud layer. Due to energy constraints, this layer features greater computing and storage capabilities than mobile fog UAVs. Moreover, the static fog layer consists of a set of FBSs and is capable of communicating with the cloud layer.

In our model, we propose using a genetic algorithm to optimize task dispatching among FBSs and fog UAVs. The latter effectively execute multiple tasks from various concurrent drones using a simplified FIFO (First-In, First-Out) strategy.

- Cloud Layer:

The cloud layer refers to IoD data centers that provide cloud computing services, such as storage and real-time processing of the streamed data captured by the cloudlet UAVs. The data centers consist of both traditional, stationary cloud data centers (referred to as static IoD cloud) and a dynamic IoD cloud, which includes computing resources from various IoD entities, including temporarily deployed UAVs. UAVs access these IoD cloud services either by interacting with the stationary fog layer or directly. This model builds on the advantages of the traditional IoD cloud and extends its capabilities to IoD entities by integrating a temporary IoD cloud that utilizes IoD computing resources, enhancing the established GA BS-Fog model.

### 3.3. GA Hybrid-Fog Formulation

Task offloading to various fog nodes (FUAVs and FBSs) and task scheduling are the most crucial challenges in IoD networks. To address the first challenge, we have proposed a hybrid fog architecture, which is based on Algorithm 1. In particular, we propose the use of a GA to provide an optimal task-offloading solution, minimizing both transmission and fog computing delays. To address the second challenge, we have used a scheduling algorithm (Algorithm 2), which calculates the transmission and computing delays of each task. It then determines the maximum delay across all tasks allocated to a single processing station [28].

- GA Hybrid-Fog Channel Model

**Figure 2:**
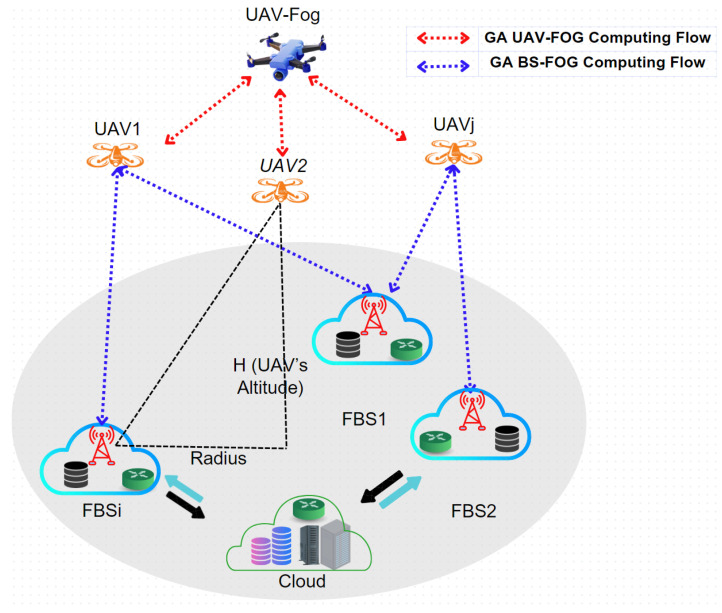
Channel model used in the GA Hybrid-Fog algorithm.

Figure 2 provides a representation of the channel model used in the GA Hybrid-Fog algorithm. For the purpose of modeling the channel connectivity over expansive geographic regions, the range between the aerial nodes (UAVs or fog UAVs) and ground devices (FBSs) must be calculated. To ensure uninterrupted data delivery between nodes, network quality must be maintained by calculating path loss to determine the optimal path for UAVs. The total path loss (assumed to be free space) from UAVs to ground FBSs can be computed using Equation (1) [29,30]:

(1) $$L{\left[dB\right]}_{d_{i}}=20log\left(d_{i}\right)+20log\left(f_{c}\right)+20log\left(\frac{4\pi }{c}\right)+L_{0}$$

where $d_{i}$ is the distance between $UAV_{i}$ and $FBS_{i}$ (Figure 2), $f_{c}$ is the channel frequency, *c* is the speed of light, and $L_{0}$ is the excess path loss, including LoS for direct communication and NLoS for indirect communication [20]. The path loss $L{\left[dB\right]}_{d_{i}}$ and the transmission power $Pt_{\left(j\right)}$ of the air devices $UAV_{i}$ are used to calculate the signal-to-noise ratio (SNR). Specifically, $SNR_{\left(i\right)}$ is calculated using Equation (2) [31]:

(2) $$SNR_{\left(i\right)}=\frac{Pt_{\left(j\right)}/L{\left[dB\right]}_{d_{i}}}{n_{0}}$$

where $n_{0}$ is the noise power.

Equation (3) defines the maximum transmission data rate $T_{i}$ from the air device to the ground device [31]:

(3) $$T_{i}=B.log_{2}\left(1+SNR_{\left(i\right)}\right)$$

where *B* represents the transmission bandwidth.

- GA Hybrid-Fog Computing Model

We begin by defining the vector of the data size of each task as D={D1, D2, …, Dj, …, Dn}; then, we calculate the transmission delay $Delay_{\left(i,j\right)}$ for offloading $TASK_{j}$ to $FBS_{i}$ using transmission rate $T_{i}$ according to the following equation [31]:

(4) $$Delay_{\left(i,j\right)}=\frac{D_{j}}{T_{i}}$$

The computing time of $FBS_{i}$ depends on its CPU frequency, $f_{i}$. The CPU cycles required to process each bit of data are represented by $\eta_{i}$. The computing delay of $TASK_{j}$ on $FBS_{i}$, denoted as $C_{\left(i,j\right)}$, is calculated using the following equation [31]:

(5) $$C_{\left(i,j\right)}=\frac{D_{j}.\eta_{i}}{f_{i}}$$

- Algorithmic Structure of a Standard GA

A GA is an evolutionary heuristic optimization algorithm that is used to find the optimal solution [32]. In a GA, the evolution starts from a random population that consists of a set of individuals (solutions). In each generation, the fitness of each individual within the population is evaluated, and a set of individuals is chosen from the existing population according to their fitness levels to create a new population for the next iteration. The algorithm concludes when either the maximum number of generations is reached or the population attains an acceptable fitness level. To produce a new population, a GA utilizes two key operations: mutation and crossover, which are detailed as follows:

1. Mutation: The population of individuals is encoded by a sequence of bits (named a chromosome). The mutation consists of randomly choosing one bit of this chromosome and changing its value.
2. Crossover: After selecting two individuals from a population, a random point is chosen, and their tails are subsequently crossed.

- GA Hybrid-Fog Algorithm

The proposed GA Hybrid-Fog algorithm, outlined in Algorithm 1, is executed by the UAV when offloading tasks to a set of FBSs or FUAVs. Before generating the population of individuals (SP), Step 1 involves defining the problem by establishing a fitness function. This function evaluates the total offloading delay for each FBS and FUAV, which is calculated using Algorithm 2. Following this, in Step 2.1, each individual in the population is encoded as a binary chromosome, representing the allocation of tasks to either FBSs or FUAVs. After that, Step 2.2 initializes a vector of SP individuals, each containing a fitness value calculated by the fitness function in Algorithm 2.

Step 3 selects a subset of the individuals in the population with the highest fitness values.

Step 4 applies the crossover function of the heuristic GA as follows:

- Step 4.1: Choose two chromosomes *j* and *j* + 1 when the generated random value r is lower than the crossover probability (PC).
- Step 4.2: Randomly select a crossover point that represents the order of the crossed bits of chromosome *j* and chromosome *j* + 1.
- Step 4.3: Cross the two bits of these chromosomes.

Step 5 applies the mutation function of the GA heuristic as follows:

- Step 5.1: Select one chromosome *j* when the regenerated random value r is lower than mutation probability (PM).
- Step 5.2: Randomly select a mutation point that represents the order of the mutated bit of chromosome *j*.
- Step 5.3: Change the value of the selected bit of chromosome *j*.

Finally, Steps 6 and 7 generate the minimal delay for all offloading tasks to all FBSs and FUAVs.

Algorithm 2 provides the pseudo-code used for calculating the total delay involved in offloading tasks according to the proposed GA Hybrid-Fog algorithm. This algorithm is specifically applied during Step 2.2 and Step 6 of Algorithm 1 to calculate the transmission delay and fog computing delay for all tasks offloaded to FBSs and FUAVs.

In Step 1, the algorithm calculates the data rate based on Equations (1)–(3). Subsequently, Step 2 computes both the transmission delay and the computing delay for each offloading task based on Equations (4) and (5). Finally, the algorithm determines the total task-offloading delay, which is the maximum delay among all the individual task-offloading delays.

**Algorithm 1** **GA Hybrid-Fog Algorithm**
**Inputs:**
Number of fog base stations (NFBSs), number of fog UAVs (NFUAVs),number of offloading tasks (NTasks),    number of generations (NG),population size (SP),    chromosome length (LC),mutation probability (PM),    crossover probability (PC).
**Outputs:**
Optimized task-offloading total delay for all fog base stations and all fog UAVs (Best_Cost).
**Step 1: Problem Definition**
**Step 1.1:** Define the problem as minimizing the total delay for all fog base stations and all fog UAVs.**Step 1.2:** The Fitness_Function is defined as the total delay.
**Step 2: Initialization**
**Step 2.1:** Encode each individual as a chromosome of LC bits for the initial population $P_{0}$.**Step 2.2:** Create a vector $VF_{0}$ of SP individuals containing initialized fitness values.**For** i=1: NG
**   Step 3: Selection**
   Select a sub-vector individuals $VF_{i}$ from $VF_{i-1}$ with the maximum fitness values.   **For** j=1 to $\mathrm{length}(VF_{i})-1$      **Step 4: Crossover Function**      Generate the random variable *r*.         **If** r<PC             **Step 4.1:** Select two individuals from $VF_{i}$, chromosome *j* and chromosome j+1.             **Step 4.2:** Choose a crossover point (the order of chromosome *j* bit and of             chromosome j+1 that will be crossed).             **Step 4.3:** Apply the crossover function on chromosome *j* and j+1.         **end**
      
**      Step 5: Mutation Function**
      Generate a random variable *r*.         **If** r<PM             **Step 5.1:** Select an individual $VF_{i}$, chromosome *j*.             **Step 5.2:** Choose mutation point (the order of chromosome *j* that will be changed).             **Step 5.3:** Apply the mutation function on chromosome $j_{in}$ selected mutation point.         **end**   **end**   **Step 6: Evaluation**   Evaluate the Fitness_Function for each individual in population *i*.   **Step 7: Update**   Update the local best for each individual in population *i*.   Update the global best for all individuals in population *i*.
**end**


**Algorithm 2** **Calculating the total delay of the offloading tasks to a single fog base station or fog UAV**
**Inputs:**
Task size vector (*D*),   radius (*R*),    channel frequency ($f_{c}$),    transmission bandwidth (*B*),    altitude of the UAV (*H*),    fog BS CPU cycles (η),    fog BS CPU frequency ($f_{i}$),    UAV transmission power ($P_{t}$),    noise power ($n_{0}$)**Output:** Total task-offloading delay (Total_Delay)
**Steps:**

**Step 1: Calculate the transmission Rate (*T*):**
Step 1.1: Determine the range of the communication:lambda = physconst(‘LightSpeed’)/$f_{c}$Range = round(hypot(H, Radius));Step 1.2: Calculate the transmission packet loss (PL), signal-to-noise ratio (SNR), and transmission data rate (*T*):PL = fspl(Range, lambda)SNR = (P_t / PL)/n_0*T* = *B* · log_2(1 + SNR)
**Step 2: Calculate Total Delay (Total_Delay):**
Step 2.1: Compute transmission delay (delay) and fog computing delay (*C*):delay = D/T,*C* = *D* · η /f_iStep 2.2: Compute total transmission delay ($ToT_{trans}-Delay$) and total fog computing delay ($ToT_{comp}-Delay$):$ToT_{trans}-Delay$ = Delay(1);$ToT_{comp}-Delay$ = C(1);   **for** i = 1: (Length(D) − 1)      $ToT_{trans}-Delay$ = $ToT_{trans}-Delay$ + Delay(i + 1);      $ToT_{comp}-Delay$ = $ToT_{comp}-Delay$ + C(i);      **if** ($ToT_{trans}-Delay$ > $ToT_{comp}-Delay$)            $ToT_{comp}-Delay$ = $ToT_{trans}-Delay$;      **end**   **end**Total_Delay = $ToT_{comp}-Delay$ + C(length(D));

## 4. Numerical Results

This section presents the configuration of our simulation and a comparison of the results between our proposed GA Hybrid-Fog and different IoD technologies integrated with a GA (GA BS-Fog, GA UAV-Fog, and GA UAV-Edge).

**Simulation and parameter settings:**

We conducted our simulation using the MATLAB R2016a toolbox in order to evaluate the performance of GA Hybrid-Fog for task offloading in IoD networks. Table 2 shows the simulation parameter settings, in which three types of devices were deployed: BS-Fog, UAV-Fog, and UAV-Edge.

Our simulation consisted of two phases:

**Phase 1:** We evaluated the proposed GA Hybrid-Fog algorithm by comparing it with two existing task-offloading methods:

- **Particle Swarm Optimization (PSO)**: Designed for static fog task offloading, as proposed in [13].
- **Mixed-Integer Linear Programming (MILP)**: Introduced in [14] for mobile fog task offloading.

**Phase 2:** We compared the GA Hybrid-Fog solution with the following integrated IoD technologies:

- **GA UAV-Edge:** Uses a GA for task distribution between UAVs in the edge layer.
- **GA BS-Fog:** Uses a GA for task offloading to fog BSs.
- **GA UAV-Fog:** Uses a GA for task offloading to fog UAVs.
- **GA Hybrid-Fog:** The proposed solution, which uses a GA for task offloading to both fog BSs and fog UAVs.

**Figure 3:**
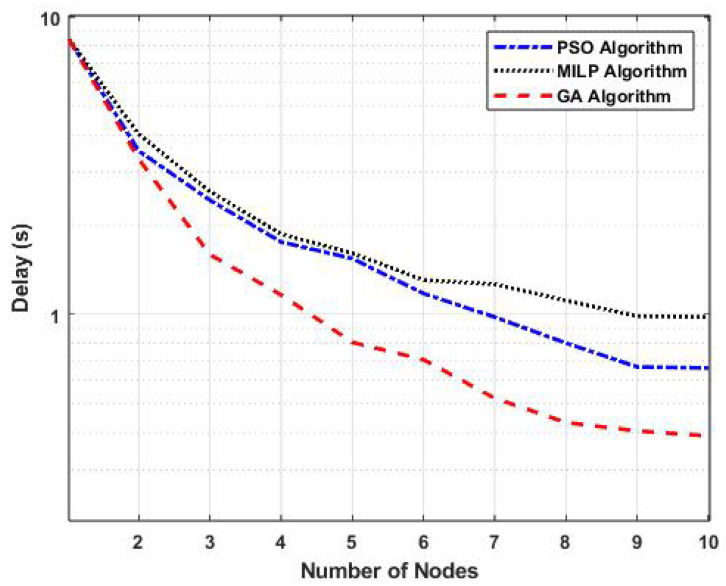
Performance comparison of task-offloading methods based on the number of nodes.

Figure 3 presents a performance comparison of task-offloading methods based on the number of nodes, with the number of offloaded tasks fixed at 200. The figure illustrates the variations in task-offloading delay (in seconds) for our proposed GA algorithm, as well as the PSO and MILP algorithms, as a function of the number of fog nodes.

As shown, increasing the number of fog nodes reduced task-offloading delay across all methods. This decrease is attributed to the enhanced processing capacity provided by additional nodes. The results indicate that GA had a lower delay compared to PSO and MILP. Specifically, when the number of nodes was seven, the task-offloading delay using GA was 0.52 s, whereas PSO resulted in a delay of 0.97 s and MILP incurred a delay of 1.26 s. This corresponds to reductions in delay of 46.39% compared to PSO and 58.73% compared to MILP.

**Figure 4:**
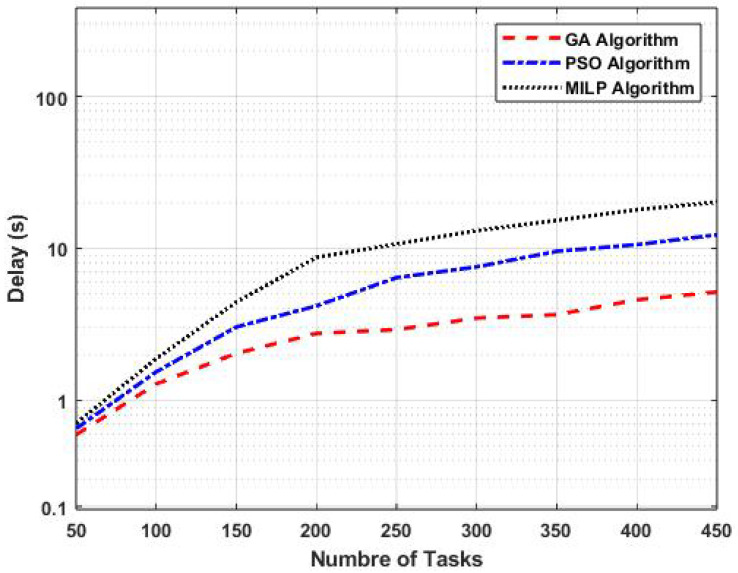
Performance comparison of task-offloading methods based on the number of tasks.

Figure 4 presents a performance comparison of task-offloading methods based on the number of tasks. The figure illustrates the variations in task-offloading delay for our proposed GA algorithm, as well as the PSO and MILP algorithms, as a function of the number of tasks, with the number of fog nodes fixed at four.

As shown, an increase in the number of tasks resulted in a higher task-offloading delay across all methods. This is due to the additional computational demand imposed on the fog nodes. The results indicate that GA had a lower delay compared to PSO and MILP. Specifically, for 200 tasks, the task-offloading delay using GA was 2.7 s, whereas PSO resulted in a delay of 4.1 s and MILP incurred a delay of 8.7 s. This corresponds to reductions in delay of 34.15% compared to PSO and 68.97% compared to MILP.

Furthermore, Figure 3 and Figure 4 highlight that the GA Hybrid-Fog approach consistently outperformed the other task-offloading methods. This advantage is attributed to the genetic algorithm’s efficient exploration and optimization capabilities, which make it particularly well suited for dynamic environments.

**Figure 5:**
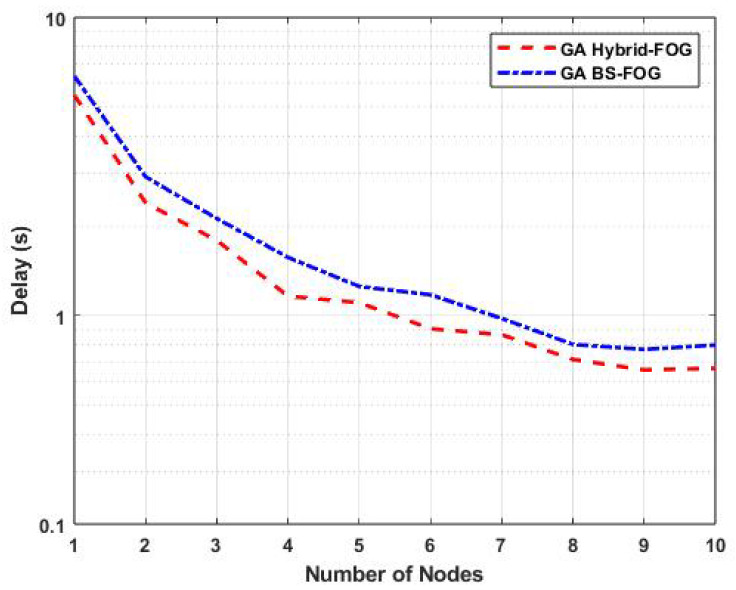
Variations in task-offloading delay as a function of the number of tasks for various IoD technologies.

Figure 5 displays the task-offloading delay (in seconds) for our proposed GA Hybrid-Fog approach and the GA BS-Fog approach as a function of the number of nodes (FBSs and/or FUAVs), which varied from 1 to 10, with the number of offloaded tasks fixed at 100.

Specifically, for five nodes, the task-offloading delay using GA Hybrid-Fog was 1.1 s, whereas GA BS-Fog had a delay of 1.25 s. This represents an improvement of 12% when using GA Hybrid-Fog compared to GA BS-Fog. Furthermore, the figure highlights that the GA Hybrid-Fog approach had a consistently lower delay compared to GA BS-Fog. This is because GA BS-Fog relies solely on BSs, whereas the GA Hybrid-Fog approach utilizes both FBSs and FUAVs. Additionally, the GA Hybrid-Fog algorithm employs a heuristic optimization method to produce an optimal task-offloading solution, thereby minimizing delay for both task transmission and processing.

**Figure 6:**
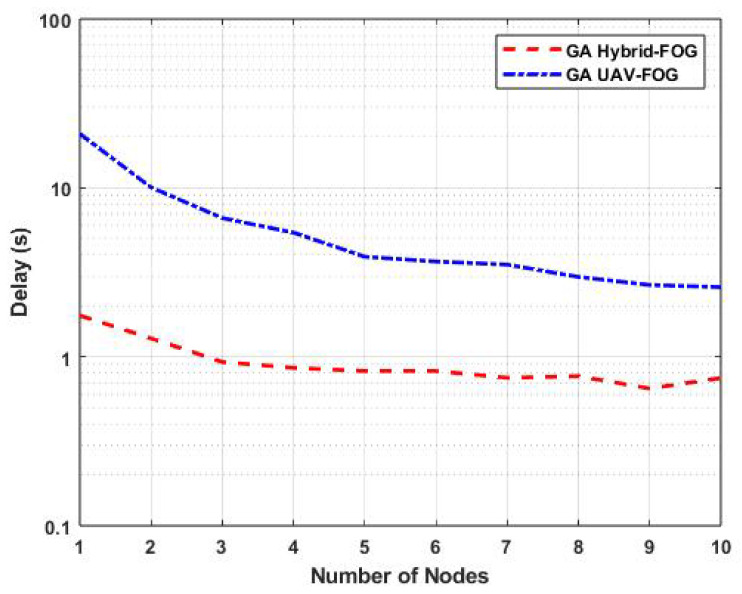
Variations in task-offloading delay as a function of the number of nodes for GA Hybrid-Fog and GA UAV-Fog.

Figure 6 illustrates the task-offloading delays of the proposed GA Hybrid-Fog approach and the GA UAV-Fog approach as a function of the number of nodes, with the number of fog BSs fixed at five. Similar to the previous results, increasing the number of nodes led to a decrease in task-offloading delay for both the GA Hybrid-Fog and GA UAV-Fog methods. Specifically, for five nodes, GA Hybrid-Fog had a task-offloading delay of 0.8 s, whereas GA UAV-Fog had a delay of 3.9 s. This represents a significant improvement of 79.49% when using GA Hybrid-Fog compared to GA UAV-Fog. Additionally, Figure 6 demonstrates that our proposed GA Hybrid-Fog consistently had a lower task-offloading delay compared to GA UAV-Fog. This improvement is attributed to the higher capacity of our proposed protocol, which leverages both FUAVs and FBSs, in contrast to GA UAV-Fog, which relies solely on FUAVs.

**Figure 7:**
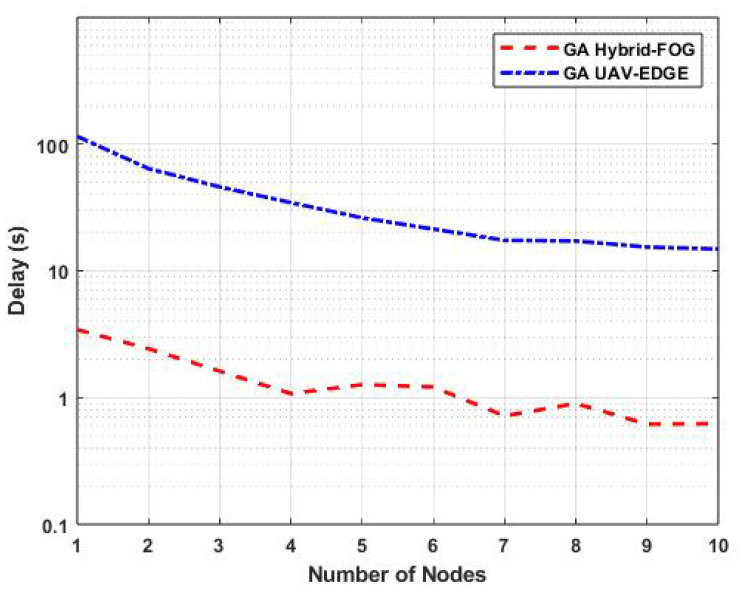
Variations in task-offloading delay as a function of the number of nodes for GA Hybrid-Fog and GA UAV-Edge.

The task-offloading delay of the proposed GA Hybrid-Fog approach and that of GA UAV-Edge as a function of the number of nodes (number of FUAVs or number of edge UAVs) is shown in Figure 7, with the number of offloaded tasks fixed at 100. The results show that GA Hybrid-Fog outperformed GA UAV-Edge, with a significantly lower offloading delay. Specifically, for five nodes, GA Hybrid-Fog had a delay of 1.3 s, whereas GA UAV-Edge experienced a delay of 26.2 s, representing a 95.04% improvement. This confirms the superiority of GA Hybrid-Fog, which benefits from the combined processing capacity of both FBSs and FUAVs, unlike GA UAV-Edge, which relies solely on edge UAVs.

**Figure 8:**
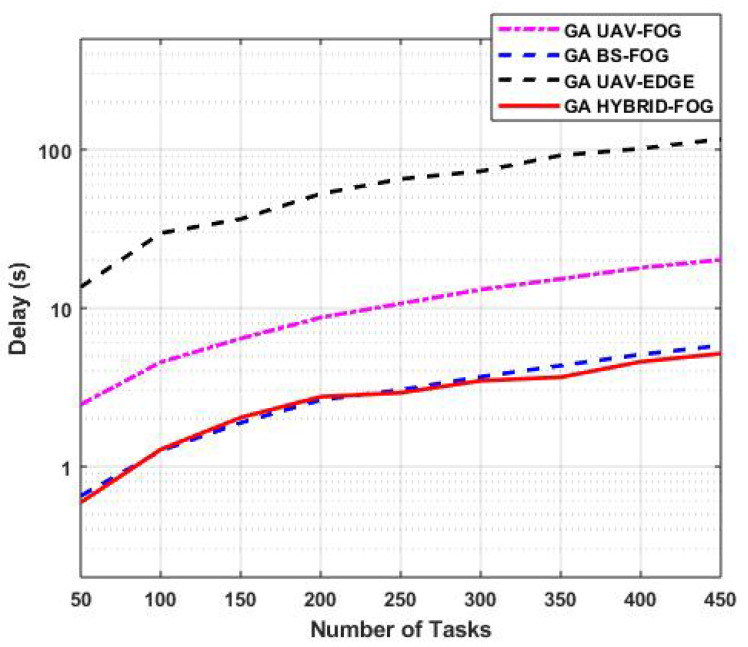
Variations in task-offloading delay with respect to the number of tasks for various IoD technologies.

Figure 8 presents the variations in task-offloading delay with respect to the number of tasks (TN) for various IoD technologies, including GA Hybrid-Fog, GA BS-Fog, GA UAV-Fog, and GA UAV-Edge, where the number of FUAVs and FBSs was fixed at five. GA Hybrid-Fog exhibited the lowest offloading delay compared to the other IoD technologies. For a TN of 350 tasks, GA Hybrid-Fog resulted in a delay of 3.6 s, GA BS-Fog had a delay of 4.3 s, GA UAV-Fog experienced a delay of 15.3 s, and GA UAV-Edge had a delay of 92.1 s. This is because the proposed GA Hybrid-Fog algorithm utilizes both FUAVs and FBSs, leveraging a total of 10 nodes compared to just 5 in the other configurations.

In terms of improvement, GA Hybrid-Fog achieved a 16.28% improvement over GA BS-Fog, a 76.47% improvement over GA UAV-Fog, and a 96.09% improvement over GA UAV-Edge. These results highlight the significant reduction in offloading delay achieved by the GA Hybrid-Fog algorithm compared to the other IoD technologies.

**Figure 9:**
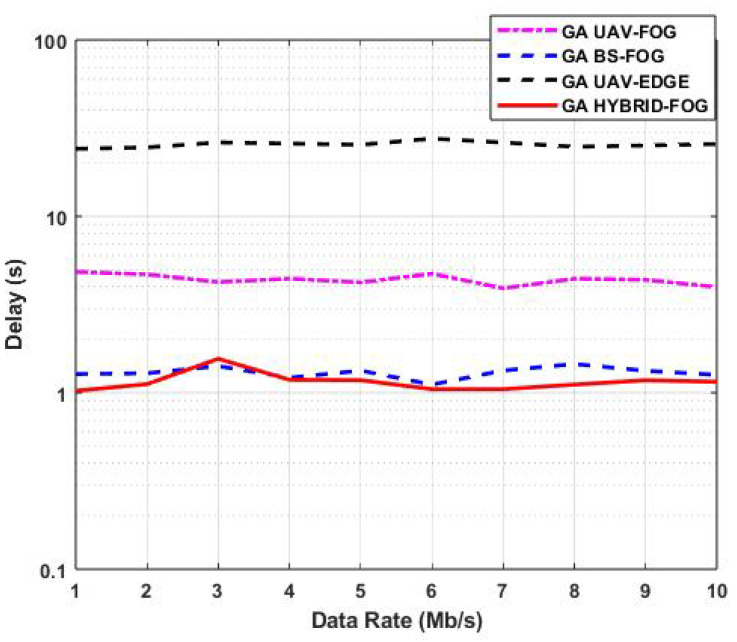
Variations in task-offloading delay as a function of the data rate for various IoD technologies.

Figure 9 illustrates the variations in task-offloading delay as a function of the data rate (DR) for various IoD technologies, where the number of devices was fixed at five for both NFBSs and NFUAVs. The variations in the DR reflect changes in channel conditions, as shown in Equations (1) and (3), which primarily depend on range variation. The results demonstrate that the GA Hybrid-Fog method had a significantly lower offloading delay compared to other IoD technologies. This improvement is attributed to its efficient task distribution across fog BSs, fog UAVs, and edge UAVs.

For instance, at a DR of 5 Mb/s, the GA Hybrid-Fog method achieved the best performance with a delay of 1.1 s. This represents a 15.38% improvement over the GA BS-Fog method (1.3 s), a 73.81% improvement over the GA UAV-Fog method (4.2 s), and a remarkable 95.67% improvement over the GA UAV-Edge method (25.4 s). These results highlight the superior performance of the GA Hybrid-Fog approach in minimizing offloading delays across various scenarios.

## 5. Conclusions

In this paper, we addressed the problem of task offloading in IoD networks by proposing the GA Hybrid-Fog algorithm, which integrates a genetic algorithm with a hybrid fog computing architecture. The experimental results demonstrated the effectiveness of our approach in minimizing task-offloading delay across different network conditions.

We evaluated the proposed GA Hybrid-Fog algorithm by comparing it with two existing task-offloading methods, PSO and MILP. The results showed that the GA Hybrid-Fog algorithm consistently outperformed the other task-offloading methods. For instance, in specific scenarios, the GA Hybrid-Fog algorithm achieved a 46.39% improvement over PSO and a 58.73% improvement over MILP.

Beyond these improvements, additional evaluations demonstrated the superiority of GA Hybrid-Fog over other task-offloading architectures. The results showed that the proposed architecture achieved significantly lower delays compared to GA BS-Fog, GA UAV-Fog, and GA UAV-Edge. For instance, in specific scenarios, GA Hybrid-Fog achieved a 16.28% improvement over GA BS-Fog, a 76.47% improvement over GA UAV-Fog, and a 96.09% improvement over GA UAV-Edge in task-offloading delay.

By varying the number of tasks, we showed that the proposed GA Hybrid-Fog algorithm consistently outperforms other task-offloading technologies. Furthermore, through the analysis of variations in range and channel conditions, we observed that by leveraging both the genetic algorithm and a hybrid architecture, the GA Hybrid-Fog algorithm delivered substantial improvements in task-offloading delay.

As a future research direction, we plan to incorporate a UAV energy consumption model into our study, exploring the interplay between environmental factors and algorithm performance. Moreover, we intend to apply the proposed GA Hybrid-Fog approach to real-time experiments to validate its performance in practical scenarios. This will enable us to develop a more comprehensive framework for evaluating the sustainability and efficiency of our approach.

## Figures and Tables

**Table 1:** A comparative analysis of IoD fog computing studies.

Study	Nature of Fog Node(s)	Objective(s)	Addressed Problem(s)	Optimization Technique(s)	Application Domain
(S. Zaidi et al., 2025) [13]	Stationary	Real-time latency	Task offloading	Particle swarm optimization (PSO)	General
(M. Aldossary, 2024) [14]	Mobile	Real-time latency, energy consumption	Task offloading	mixed-integer linear programming (MILP)	General
(G. Sun et al., 2024) [15]	Mobile	System utility, real-time latency, energy consumption	Task offloading, resource allocation	Game-theoretic, convex optimization, evolutionary computation	Post-disaster rescue
(F. S. Abkenar, 2022) [16]	Mobile	Real-time latency, energy consumption	UAV deployment, task offloading, wireless power transfer (WPT)	Convex optimization	Disaster management
(W. Min, 2023) [17]	Mobile	Real-time latency, service availability, cost of resources	Task offloading	Dynamic programming	General
(J. Yao and N. Ansari, 2022) [18]	Stationary	Real-time latency, energy consumption	Task offloading, power control	Convolutional neural network (CNN), mixed-integer nonlinear programming (MINLP)	Object recognition
(Q. Zhang et al., 2022) [19]	Stationary	Service availability	Task offloading	Stochastic Reward Nets (SRNs)	General
(S. Tong et al., 2022) [20]	Stationary	Energy consumption	Task offloading, resource allocation, UAV deployment	MINLP	General
(A. Naouri, 2024) [21]	Mobile	Low computational complexity	Connectivity, coverage, UAV deployment	One-dimensional swapping method, Adaptive Whale Optimization Algorithm (WOA)	Critical rescue operations
Our proposal	Hybrid	Real-time latency	Task offloading	Genetic algorithm (GA)	General

**Table 2:** Simulation parameters.

Parameter	Value
Radius	500
Transmission bandwidth (B)	0.2 MHz
Number of fog BSs (NFBSs)	[1, …, 10]
Number of fog UAVs (NFUAVs)	[1, …, 10]
UAV operating altitude (H)	360 m
Number of tasks (Ntasks)	[50, …, 450]
Fog BS CPU cycles (η)	1000 cycle/bit
Fog UAV CPU cycles (η)	100 cycle/bit
Edge UAV CPU cycles (η)	50 cycle/bit
UAV transmission power (Pt)	10 dBm
Fog BS CPU frequency ($f_{i}$)	$2.4\times 10^{9}$ cycle/s
Fog UAV CPU frequency ($f_{i}$)	$2.4\times 10^{7}$ cycle/s
Edge UAV CPU frequency ($f_{i}$)	$2.4\times 10^{7}$ cycle/s
Noise power ($n_{0}$)	−105 dBm
Number of generations (NG)	250
Population size (SP)	150
Chromosome length (LC)	40 bits
Mutation probability (PM)	6.25%
Crossover probability (PC)	80%

## Data Availability

The dataset is available on request from the authors.
